# Evolution of thymopoietic microenvironments

**DOI:** 10.1098/rsob.200383

**Published:** 2021-02-24

**Authors:** Ryo Morimoto, Jeremy Swann, Anja Nusser, Inês Trancoso, Michael Schorpp, Thomas Boehm

**Affiliations:** Department of Developmental Immunology, Max Planck Institute of Immunobiology and Epigenetics, Stuebeweg 51, 79108 Freiburg, Germany

**Keywords:** vertebrate, adaptive immunity, antigen receptor, thymus, Foxn1, self-tolerance

## Abstract

In vertebrates, the development of lymphocytes from undifferentiated haematopoietic precursors takes place in so-called primary lymphoid organs, such as the thymus. Therein, lymphocytes undergo a complex differentiation and selection process that culminates in the generation of a pool of mature T cells that collectively express a self-tolerant repertoire of somatically diversified antigen receptors. Throughout this entire process, the microenvironment of the thymus in large parts dictates the sequence and outcome of the lymphopoietic activity. In vertebrates, direct genetic evidence in some species and circumstantial evidence in others suggest that the formation of a functional thymic microenvironment is controlled by members of the Foxn1/4 family of transcription factors. In teleost fishes, both *Foxn1* and *Foxn4* contribute to thymopoietic activity, whereas *Foxn1* is both necessary and sufficient in the mammalian thymus. The evolutionary history of *Foxn1/4* genes suggests that an ancient *Foxn4* gene lineage gave rise to the *Foxn1* genes in early vertebrates, raising the question of the thymopoietic capacity of the ancestor common to all vertebrates. Recent attempts to reconstruct the early events in the evolution of thymopoietic tissues by replacement of the mouse *Foxn1* gene by *Foxn1*-like genes isolated from various chordate species suggest a plausible scenario. It appears that the primordial thymus was a bi-potent lymphoid organ, supporting both B cell and T cell development; however, during the course of vertebrate, evolution B cell development was gradually diminished converting the thymus into a site specialized in T cell development.

## Graphical abstract

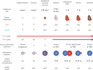

## 1. Introduction

Adaptive immune systems of vertebrates rely on the formation and expression of randomly generated antigen receptor repertoires in their lymphocyte lineages. As a result, the human immune system for instance generates a large number of lymphocyte clones each producing a unique antibody or T cell receptor (TCR) to specifically recognize a diverse array of antigens. Remarkably, the facility of whole-sale somatic diversification of antigen receptors was invented twice, once in the lineage leading to extant jawless fishes, and a second time in the lineage leading to extant jawed vertebrates [1]. Interestingly, the mechanisms by which functional antigen receptors are assembled, as well as the structures of the respective building blocks of their receptors differ between the two branches of vertebrates. The variable lymphocyte receptors (VLRs) of jawless vertebrates consist of tandemly arranged leucine-rich repeats (LRRs) [2,3]. They are assembled from a large number of individual LRR-encoding genomic cassettes through the action of specialized cytidine deaminases, in a process akin to gene conversion [4,5]. By contrast, the basic structural unit of the somatically diversified antigen receptors of jawed vertebrates is the immunoglobulin fold [6]; *immunoglobulin* (*Ig*) and *TCR* genes are assembled into functional units from variable, diversity and joining elements by the products of recombination activating genes (RAG) 1 and 2 [7,8]. The RAG enzymes recognize evolutionarily conserved recombination signal sequences [9] to direct the orderly succession of site-specific DNA cutting and joining processes. The apparent discrepancy of antigen receptor structures and assembly mechanisms leaves unanswered the question of whether or not the common ancestor of vertebrates already possessed a system for somatic diversification: did it consist of one of the two extant forms, or perhaps even an alternative—now extinct—system? Be this as it may, the vast diversity of somatically generated antigen receptors underlying the adaptive immune systems of vertebrates raises the question as to how the repertoire can be purged of self-destructive reactivities—and thus ignore self—yet retain the capacity to recognize non-self or altered self-structures. The evolutionary solution to this problem has been the emergence of specialized lymphoid organs, wherein differentiation of lymphocytes, the formation of functional antigen receptors, and the subsequent quality control of the emerging receptor repertoire are spatially coupled [10]. Indeed, these coordinated activities are the raison d́être of so-called primary lymphoid organs such as the thymus. Like other haematopoietic sites that are home to developing lymphocytes, the microenvironment of the thymus not only provides a supportive environment that fosters attraction and differentiation of lymphocytes, but also orchestrates the complex steps required to achieve a highly diverse yet self-tolerant repertoire of antigen receptors expressed by mature effector cells [11].

Recent excellent reviews have discussed the cellular heterogeneity and function of individual components of the thymic microenvironment [12–14]; moreover, the various signalling pathways that instruct differentiation of haematopoietic precursor cells to enter the T cell lineage [15] and the components controlling the chemotactic movements of lymphocytes within the thymus [16,17] have been extensively reviewed. Likewise, the function of several regulators that allow the stroma to execute the quality control of antigen receptors, including the process of promiscuous gene expression [18,19], has been well documented. While briefly referring to the main findings in this area, our focus here is on the evolutionary history of the thymic microenvironment. In order to provide a perspective for this discussion, we examine the mechanisms by which antigen receptors in jawless and jawed vertebrates are assembled into functional units, to emphasize the degree of diversity that is generated by the process of somatic diversification. We then touch upon the general structure of the thymic microenvironment in order to highlight its role in supporting the development of T cells and the associated quality control of the TCR repertoire for self-compatibility. This summary sets the stage for a discussion of the evolutionary history of a transcription factor family whose diversification appears to be directly coupled to the changes in the lymphopoietic activity of the thymus during the approximately 500 million years of vertebrate evolution.

## 2. Somatic diversification of vertebrate antigen receptors

Jawless vertebrates comprise a small group of species of lampreys and hagfishes; they are the sister group of the much more species-rich branch of jawed vertebrates [20]. In their alternative adaptive immune system [21], non-functional germ-line components of the three known VLR genes are assembled into complete units by specialized cytidine deaminases (CDA) [5]; CDA2 is known to be required for assembly of the *VLRB* gene [22], and circumstantial evidence implicates CDA1 and its relatives in the assembly of *VLRA* and *VLRC* genes [5,23–25]. The evolutionary history of cytidine deaminases suggests that an ancient anti-parasite defense system based on the nucleotide-specific mutation of foreign DNA was re-purposed to create a more sophisticated and indirect immune surveillance system [25,26]. Instead of indiscriminately mutating cytidines in the DNA of genetic parasites (such as retroviruses), domesticated cytidine deaminases target the genes encoding antigen receptors in host genomic DNA [26]. In this manner, cytidine deaminases contribute to a self-/non-self-discrimination system with increased target range. The risk associated with potentially disastrous pan-genomic alterations caused by the activities of self-mutators is minimized by mechanism(s) that evolved to confine mutagenic activity to (i) specific locations in the genome and (ii) particular stages during the differentiation of immune effector cells. It is conceivable that once the targets for cytidine deaminases in the host genome had become established, combinatorial assembly of the subgenomic fragments enabled the generation of a vast array of different VLRs. It should be noted that the activity of cytidine deaminases is not restricted to the immune system of jawless fishes, since a related enzyme, activation-induced cytidine deaminase (Aicda) is involved in the immune response-related diversification of antibody genes in jawed vertebrates [27]; in cartilaginous fishes, this may also apply to *TCR* genes [28,29]. The principal building blocks of VLRs are leucine-rich repeats (LRRs) of 24 amino acid residues each that are assembled into tandem arrays of different lengths, rarely (if ever) involving junctional diversity at their fusion sites [1,2]. This observation indicates that the generative mechanism of VLR repertoires rests on combinatorial diversity; nonetheless, because hundreds of different LRR cassettes are encoded in the genome, and the receptors may contain different numbers of cassettes, combinatorial diversification can generate billions of different receptors [30–32]. The paucity of junctional diversification in VLRs is supported by the apparent lack, in the genome of jawless vertebrates, of a homologue of terminal deoxynucleotidyltransferase (TdT). TdT is a close relative of DNA polymerase mu [33] that is a member of the X family of polymerases involved in DNA repair [34]. In the immune system of jawed vertebrates, TdT is specifically deployed to add non-templated (random) nucleotides to the coding ends of variable (V), diversity (D) and joining (J) regions during assembly of *Ig* and *TCR* genes of jawed vertebrates [35]. This mechanism considerably increases their sequence diversity in addition to combinatorial assembly, which—owing to the comparatively small number of genetic elements [36]—is more limited than that of VLR assemblies [1].

In jawed vertebrates, RAG1 and RAG2 fuse *Ig* and *TCR* genes into functional units [37]. Recombination relies on the presence of specialized recombination signal sequences at the ends of V, D, J regions that dictate a stereotyped assembly process; in the case of *TCRA*, *TCRG*, *IGL* and *IGK* recombination generates a V-J fusion, whereas in the case of *TCRB*, *TRCD* and *IGH* V-D-J fusions are generated (for *TCRD*, more than one D element might be included in the final product). As a result of a rather strict assembly rule, combinatorial diversity is constrained and can, at most, generate a few thousand complete variable parts of Ig or TCRs. Hence, the addition of non-templated nucleotides by TdT is paramount to increasing the diversity of the antigen-binding sites of the resulting *Ig* and *TCR* genes [38,39].

In the case of RAG proteins, recent studies reconstructed, in exquisite molecular detail, how the transposases that were part of ancient genetic parasites became domesticated and turned into self-DNA mutators subserving the needs of their host [40,41]. Of note, *RAG*-like genes are also found in the genomes of many invertebrates, such as the purple sea urchin *Strongylocentrotus purpuratus* which exhibits *RAG1*-like and *RAG2*-like sequences [42]. Most interestingly, the genome of the basal chordate amphioxus *Brachiostoma belcheri* possesses a transposable element designated *ProtoRAG*, which encodes both RAG1-like and RAG2-like proteins; its configuration is equivalent to the structure of the presumptive ancestral RAG transposon [43]. Although these observations are compatible with a vertical rather than horizontal transmission mode of *RAG*-like sequences in the deuterostome and chordate lineages [44], it should be noted that such sequences have not been found in the genomes of extant jawless fishes. The conversion of the ancestral Transib transposase into the heterodimeric RAG recombinase appears unlikely to have occurred in a single step. Rather, it appears that a Transib transposon at some point acquired a RAG2-like sequence to form the primordial RAG1-RAG2 transposon [45,46]. This step initiated a coevolutionary process that led to the suppression of transposition in favour of coupled cleavage and recombination that is required for *Ig* and *TCR* assembly.

In conclusion, it appears that vertebrates successfully achieved domestication of DNA mutating enzymes and overcame the potential challenges to host genome integrity [47]; the emerging facility for site-directed programmed genome engineering enabled vertebrates to generate receptors with an essentially unlimited capacity of probing chemical space. Yet, the diversification of putative antigen receptors is not unique to vertebrates. Several invertebrate species have been described where diversification arises from expansions of gene families [48,49], alternative splicing [50,51], possibly also from the variegated expression of individual receptor isotypes and potential somatic diversification [52–54]. Hence, it remains unclear why almost all vertebrate species rely on whole-sale somatic diversification rather than the evolutionarily older solutions. The exposure to rapidly evolving pathogens may explain this difference, but it could also be related to the more complex body plan of vertebrates and the associated higher demands for biosynthetic capabilities that could only be met by mutualistic interactions with a more species-rich microbiome.

## 3. Clonal expression of somatically diversifying receptor systems

The somatically diversifying antigen receptors of vertebrates are clonally expressed by lymphocytes that may have had their evolutionary origin in lymphocyte-like cells; such cells were recently described in tunicates, the chordate lineage most closely related to vertebrates [55]. It is not known whether the facility of mono-allelic expression of genes, as a pre-requisite of clonal expression of assembled genes in a diploid genome, was introduced into the lymphocyte lineage at the time of the emergence of somatic diversification. However, allelic exclusion/clonal expression appears to have evolved in a step-wise fashion. It is conceivable that a variegated expression mode of germ-line encoded pattern recognition receptors, akin to the expression of natural killer receptors in extant NK cells [56], may have preceded the expression of a single receptor as a general characteristic of vertebrate lymphocytes [57]. One possible explanation for the emergence of mono-allelic expression of somatically diversifying antigen receptors in T cells is the need to maximize the precision of immunological memory formation, such that only clones expressing the most specific receptors would be maintained in the memory pool. Otherwise, the metabolic cost incurred by maintaining a diverse pool of inferior memory cells could become prohibitively large with increasing numbers of immune responses experienced by the individual.

## 4. Self-compatibility of antigen receptors

The emergence of somatically diversifying antigen receptors afforded the immune system with the means to explore an extraordinarily complex chemical space during self-/non-self-discrimination. With respect to potential self-reactivity, two possible non-mutually exclusive outcomes can be envisaged after somatic diversification. In the case where combinatorial assembly is the norm, such as with *VLR* genes, the individual cassettes may have been selected for self-compatibility over evolutionary time [57]. Hence, even if expressed as tandem arrays, the probability of self-reactivity of VLRs may be small. In the case where the antigen-binding sites are partly composed of non-templated sequences, such as in the CDR3 regions of antibodies and TCRs, a certain degree of self-reactivity is unavoidable and self-destruction may ensue. The ability of antibodies to even discriminate stereoisomers illustrates the astounding diversity of interaction surfaces of antigen receptors [58]. How, then, can an undesired immune response against self-structures be prevented? The general solution to this problem that was implemented by vertebrates comes from the spatial association of generation and the subsequent quality control of the emerging antigen receptor repertoire [19]. Hence, primary lymphoid organs orchestrate several interconnected steps: the generative function of the thymus ensures that large numbers of lymphocytes are produced; only this ensures that enough cells eventually survive the subsequent selection for self-compatibility. In what follows, we further discuss the structure and function of the thymic microenvironment of jawed vertebrates to highlight the relative contributions of these two principal functions to thymopoiesis. Because little is known about the microenvironment of the lamprey thymoid, these results are not analysed in detail in the comparative discussion here.

## 5. Location, anatomy and development of thymic microenvironments

Whereas the anatomical site of general haematopoietic tissues is very variable in vertebrates [10], thymopoietic microenvironments are always located in the pharynx. Throughout vertebrate evolution, the epithelial component of thymic stroma develops from pharyngeal endoderm [11,57] and may have its origin in a circumscribed patch of pharyngeal epithelium that was identified in developing larvae of the cephalochordate amphioxus [59]. The chordate foregut is known for its developmental flexibility and propensity for morphological innovations and hence may have served as a fertile ground for the emergence of a primordial lymphopoietic structure [10,60]. This tissue may have later acquired additional functions, for instance in the quality control process of antigen receptors after the emergence of their somatic diversification. The final combination of lymphopoietic and tolerogenic facilities of the thymic microenvironment must have made this tissue so unique that its functions could not be easily re-established elsewhere in the organism, which may have contributed to the remarkable evolutionary stability of its anatomical location.

An important pre-requisite for spatial control over lymphocyte differentiation is the ability to direct the migration of haematopoietic precursor cells and their descendants to, and guide their movement within, a primary lymphoid organ. It is noteworthy that genes encoding chemokines and their receptors have not yet been found in the genomes of non-vertebrate chordate species. Although cephalochordates and urochordates both possess peptide-binding G protein-coupled receptors (GPCRs) [61,62], none of their GPCRs shows the characteristic chemokine receptor signature motif DRYLAIV within the second intracellular domain. Collectively, these observations suggest that the chemokine signalling system has its origin in the hypothetical common vertebrate ancestor. Indeed, genes for Cxcr4 and a number of other chemokine receptors and their putative ligands are present in the genomes of lampreys [59] and cartilaginous fishes [63,64], compatible with the view that they represent components of a genetic network orchestrating the programmed interaction of haematopoietic cells and dedicated environments. The Cxcr4 receptor and its ligands are considered to represent one of the most ancient representatives of the chemokine receptor/ligand gene families [65]. In studies examining the physiological role of chemokine signals emanating from the thymic epithelium, it was found that the expression of Cxcl12, a major ligand of Cxcr4, was sufficient to support the attraction of Cxcr4-expressing haematopoietic precursor cells to the thymic rudiment; the ensuing interaction with the Notch-ligand Delta-like 4 initiates T cell development [66]. This observation assigns a putative role of Cxcr4 and its ligand Cxcl12 to the regulation of thymoid homing also in lampreys.

## 6. Elements of quality control in the thymus

TCRs are expressed on the surface of T cells, and unlike the corresponding BCR on B cells, are not shed after engagement with antigen. Indeed, in jawed vertebrates, antigen recognition by T cells requires the presence of a dedicated antigen presentation mechanism centred around the intracellular proteolytic breakdown of antigen and loading onto major histocompatibility complex (MHC) molecules for exposure at the cell surface [67]. It is highly likely that T cells of jawless vertebrates recognize antigen in an analogous fashion, although the functional equivalent of MHC, if any, has not yet been identified. The evolutionary origin of MHC is unclear, although it has been speculated that it initially served in inter-individual mate choice decisions and was only later co-opted into an immunological intra-individual self-/non-self-discrimination mechanism [68].

In the thymus, T cells need to interact, via their TCRs, with self-peptide-loaded MHC complexes in order to complete their maturation process [19]. This interaction serves two ends; first, it ensures that T cells are screened for the expression of a functional TCR, without which they would not be able to interact with the peptide-MHC complexes and thus undergo ‘death by neglect', and second, it allows the repertoire to be purged of overly self-reactive antigen receptors, either by negative selection (that is, clonal deletion), or developmental re-routing of T cells into a regulatory lineage [69,70]. In order for the second step to achieve the desired outcome, it is important that the spectrum of self-antigens expressed in the thymus is as complete as possible, which is made possible through a process referred to as promiscuous gene expression [71]. One of the critical regulators of this latter process is the product of the *Aire* gene [18], although some other factors impact this process as well [72]. Collectively, thymic epithelial cells play a key role in fostering T cell development in the thymus; this ranges from attraction of progenitors, to their lineage specification, and support for their positive and negative selection, and culminates in the orchestration of the egress of fully mature T cells.

## 7. Histological character and cTEC/mTEC differentiation

Both histologically and functionally, the thymus of jawed vertebrates can be divided into two major compartments. The cortical epithelium provides the microenvironment for positive selection of immature thymocytes, whereas the medulla subsequently mediates negative selection of self-reactive T cells. The dispersed localization of the lamprey thymoids at the tips of gill filaments sharply contrasts to the compact lobular nature of the thymus of higher jawed vertebrates [73]. The thymoid of lampreys lacks such a clear histological demarcation of corticomedullar regions, and this difference has been attributed to the lack of coalescence of TEC progenitor cells in jawless vertebrates [74]. Nonetheless, the observation that cells expressing *CDA1* are located at the outer edge of the lam­prey thymoids points to a potential zonal differentiation of this tissue as well. Since VLR assembly appears to occur in immature lamprey lymphocytes, the outer zones of both thymoid and thymus may have similar roles in mediating the first steps of the T cell maturation sequence [73]. The structural differences between the two sister groups of vertebrates, particularly the absence of a prominent medullary region in the lamprey thymoid may be related to different requirements in dealing with self-reactivity. It is possible that the outcome of combinatorial diversity of the VLR system is more predictable and hence can be regulated in a simpler fashion than the outcome of non-templated junctional diversity associated with V(D)J recombination characteristic of jawed vertebrates.

## 8. The *Foxn1/4* gene family

In mammals, a single master transcription factor, forkhead box protein N1 (Foxn1) [75] converts undifferentiated patches of the pharyngeal endoderm to thymopoietic epithelia [76]. Thymic epithelial cells (TECs) of all jawed vertebrates so far investigated express *Foxn1*, suggesting that this factor plays an important role at or near the top of the hierarchy in the genetic network regulating thymus development [59]. However, it is obvious that *Foxn1* on its own cannot induce a thymopoietic phenotype in epithelial cells, since certain types of skin keratinocytes that also express *Foxn1* lack thymopoietic capacity [77]. Rather, it appears that other components relevant for the specification of pharyngeal endoderm are required to create a permissive environment for *Foxn1* to exert its thymopoietic capacity.

Foxn1 and its paralog Foxn4 constitute a two-member family of vertebrate wing-helix transcription factors that recognize a consensus GC-rich DNA target sequence [78,79]. Phylogenetic analyses indicate that these two genes are descendants of an ancestral metazoan *Foxn4*-like gene, which gave rise to lancelet *Foxn4* (designated as *Bl_Foxn4*), the only member of the *Foxn1/4* gene family found in the cephalochordate *Branchiostoma lanceolatum* genome [59]. At or shortly after the emergence of vertebrates, the ancestral *Foxn4* gene gave rise to the vertebrate-type *Foxn1* and *Foxn4* genes through a gene duplication event [59]; this event may have been part of the whole genome duplication(s) that occurred at the time of vertebrate emergence [80–82]. Foxn1/4 family proteins show high sequence similarity in the conserved DNA binding domains and all possess a C-terminal activation domain [83]. Most of the sequence differences among these proteins are found in the N-terminal regions preceding the DNA binding domain; this finding suggests that these modifications may be related to the different functional characteristics of these transcription factors [84,85].

## 9. Functional dissection of *Foxn1/4* genes

Reconstruction of early steps in the evolution of vertebrates suggests that the lineages of jawless and jawed vertebrates split about 500 million years ago [81]. No extant relatives of several key intermediate stages are known, thus complicating the resolution of important questions regarding the origins of adaptive immunity in general and of lymphoid organs in particular. In recent studies, attempts were made to resolve some of these uncertainties using a reconstruction strategy [84]. This scheme was developed considering that the general features of haematopoiesis are conserved in chordates, and that lineage decisions are strongly determined by external cues. The reconstruction thus focuses on the generation of functionally distinct thymopoietic microenvironments by replacing the cognate mouse *Foxn1* transcription factor gene with other members of the *Foxn1/4* gene family. The results of recent experiments reporting the expression of an invertebrate *Foxn4* gene and the *Foxn1* and *Foxn4* genes of a cartilaginous fish in a correct temporal and spatial manner provided unique insights into the thymopoietic capacities of primordial thymopoietic environments in the context of mouse haematopoiesis [85]. Below, we discuss the implications of these experiments for the reconstruction of the early steps of evolution of the thymus.

## 10. A pre-adaptive thymopoietic environment

Lancelets lack an adaptive immune system, although evidence for the presence of lymphocyte-like cells was reported [86]; since no somatic diversification system seems to be present in lancelets, these cells may represent innate-like lymphocytes. Interestingly, the *Foxn4* gene of *Branchiostoma lanceolatum*, *Bl_Foxn4,* is expressed in the pharyngeal endoderm [59], possibly foreshadowing the site of a lymphopoietic environment in vertebrates. Surprisingly, when expressed in mouse TECs instead of the endogenous *Foxn1* gene, *Bl_Foxn4* exhibits low but detectable thymopoietic activity. It supports the attraction of haematopoietic progenitors and their differentiation, but only until the CD4^+^CD8^+^ double-positive (DP) stage. In the context of the present discussion, it is noteworthy that although thymocytes at the DP stage express TCRs, they have not yet undergone any selection for antigen reactivity. In the light of the evolutionary trajectory of adaptive lymphocytes, these findings suggest that an invertebrate version of the *Foxn1/4* gene family transforms the pharyngeal endoderm into a purely lymphopoietic environment that, however, lacks the capacity for antigen receptor selection. Interestingly, the high level of *Dll4* expression induced by *Bl_Foxn4* in this reconstructed primordial form of thymopoietic environments favours T cell development and effectively suppresses B cell development. In earlier reconstitution experiments, the expression of Cxcl12 (the ligand of Cxcr4) and the Notch-ligand Dll4 in *Foxn1*-deficient TECs was sufficient to jump-start T cell development, which proceeded until the DP stage [66]. Collectively, this particular phenotype mimics the presumed requirements for a pre-adaptive (primordial) lymphocyte population that expresses germ-line encoded antigen receptors, which were selected for self-compatibility on Darwinian timescales.

## 11. Reconstructing the thymopoietic environment of a basal jawed vertebrate in mice

A peculiar feature of TECs in cartilaginous and bony fishes is the co-expression of *Foxn1* and *Foxn4* paralogs [84]. This suggests that, after the gene duplication event that gave rise to *Foxn1* from the primordial *Foxn4* gene, *Foxn1* maintained the key regulatory elements that are required for TEC expression. In reconstructions [85], the activities of these two genes were first artificially separated. It was hypothesized that the expression of the *Foxn4* gene of *Callorhinchus milii* (*Cm_Foxn4*) alone would best approximate the immunogenomic constellation after the emergence of a vertebrate form of *Foxn4*, but before the gene duplication; conversely, the expression of *Cm_Foxn1* would allow one to examine the thymopoietic qualities of an ancient relative of the mammalian *Foxn1* gene. This approach would also uncover qualitative differences between *Foxn4* and *Foxn1*, if any.

In *Cm_Foxn4*-driven TECs, the lymphoid cell population is distinguished by a large fraction of immature B cells that develop in the mesenchymal perivascular spaces of the thymic microenvironment. Although the absolute numbers of T cells are small, T cell differentiation proceeds normally in the epithelial environment of the reconstructed thymus. Collectively, these results are compatible with the lymphopoietic bipotency of the *Cm_Foxn4* gene-driven TEC differentiation. This feature appears to be a common characteristic of vertebrate Foxn4 transcription factors, since a similar result was previously observed in *Mm_Foxn4* transgenic thymi [84]. At present, it is unclear which factor(s) mediate the spatial segregation of T cell development in the epithelial environment and the B cell development in the mesenchymal perivascular space. *Cm_Foxn4* gene-driven TECs resemble cortical epithelia (cTECs) more than medullary TECs (mTECs) as indicated by immunohistochemistry, flow cytometry and gene expression profiling. By contrast, the TECs present in *Bl_Foxn4* transgenics resemble immature cTECs, with little evidence of mTEC-related features. When considering the phenotypes associated with the transition from *Bl_Foxn4* to *Cm_Foxn4*, the emergence of B cell poiesis in the thymus appears to be a vertebrate-specific quality. This observation raises the interesting question as to simultaneous and/or sequential emergence of the two principal lymphocyte lineages, which so far remains unresolved.

The *Foxn1* genes of cartilaginous fishes are considered to be the closest relatives of the primordial jawed vertebrate *Foxn1* gene family. Most surprisingly, despite more than 400 million years of independent evolution, the lymphopoietic profile of the *Foxn1* gene of *Callorhinchus milii* (*Cm_Foxn1*) was found to be very similar to that of mouse *Foxn1* [85]. Of note, the numbers of immature B cells were substantially lower in the *Cm_Foxn1* thymus than in the *Cm_Foxn4*-driven microenvironment. In addition, the stromal organization of the *Cm_Foxn1* thymus also resembled that of the mouse thymus with normally differentiated cTECs and mTECs in well-segregated of cortical and medullary areas. Moreover, the transcriptome of *Cm_Foxn1* TEC compartments exhibited mouse-like patterns of the gene expression, suggesting that the T cell-biased function of *Foxn1* was established in the early stages of jawed vertebrate evolution and has remained stable ever since. However, the thymus in cartilaginous fishes is characterized by co-expression of *Cm_Foxn1* and *Cm_Foxn4*. When this more physiological situation was re-created in transgenic mice, increased numbers of immature B cells were found. This observation strongly suggests that *Cm_Foxn1* synergizes with *Cm_Foxn4* to boost the efficiency of both T and B cell development. Interestingly, immature B cells still develop in the perivascular region of the thymi, resembling the situation in *Cm_Foxn4* single expressors. This peculiar lymphopoietic phenotype is mirrored in the anatomy of the thymus tissue and the cytological and transcriptomic characteristics of TECs. Collectively, the reconstructions are compatible with the view that the early vertebrate thymus functioned as a bi-lymphopoietic organ, fostering both B and T cell development in anatomically segregated regions of the thymus. Further experiments with chimaeric transcription factors [85] indicated that the central segment in the N-terminal region of the Foxn1 transcription factor confers the unique lymphopoietic properties on this paralog. Phylogenetic analyses suggest that this segment most likely arose through exon replacements in the duplicated form of the primordial vertebrate *Foxn4* gene in the early stages of vertebrate evolution.

**Figure 1.**
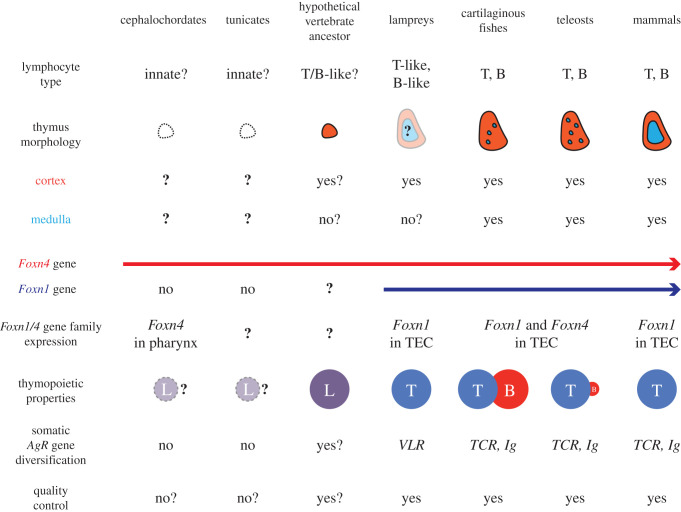
Evolution of thymopoiesis. This scheme synthesizes information relevant to the emergence of the thymus as a primary lymphoid organ in vertebrates. The character states in cephalochordates, tunicates and the vertebrate ancestor are hypothetical and are inferred from reconstruction experiments discussed in the text. With respect to thymus morphology, note that it is unclear whether the pharynx of cephalochordates and tunicates exhibits a lymphopoietic tissue (dotted lines for thymus morphology and thymopoietic properties); the orange area indicates the cortex, light blue the medulla. The symbol L refers to a hypothetical primordial lymphocyte lineage before the establishment of distinct T and B lineages. For extant vertebrates, the lineage-specific thymopoietic properties are indicated by the different sizes of circles. Quality control refers to the process of selection for self-compatibility imposed on the primary repertoire of somatically diversified antigen receptors.

Although a plausible scenario of the evolutionary trajectory of the thymic microenvironment of jawed vertebrates is emerging from these studies (figure 1), a number of important questions remain unanswered. For example, since the sequence of tunicate Foxn4 resembles, its vertebrate homologues more than that of the amphioxus protein, it will be informative to examine whether this urochordate (pre-vertebrate) form of Foxn4 has already acquired lymphopoietic bi-potency. The outcome of such an analysis would also inform the discussion of the origin of the two principal adaptive lymphocyte lineages, which remains unresolved, and may help clarify the nature of the lymphocyte-like cells of tunicates [55].

An important additional step in the evolutionary history of the *Foxn1/4* gene family is associated with *cis*-regulatory changes. It appears that *Foxn4* expression in TECs was silenced in the common ancestor of tetrapods; from then onwards, T cell development became solely dependent on the activity of *Foxn1* [75,76,87]. Perhaps, the profound immunological changes associated with the emergence of mammals necessitated the prior termination of B cell development in the thymus. In the light of the results discussed above, this phenotype could only be achieved by eliminating the activity of the pro-B factor Foxn4, and by introducing further changes to the Foxn1 protein to exclusively support T cell development. As a result, the thymus emerged as a lymphoid organ specialized in T cell development [10]; the byproduct of these events was the strict anatomical segregation of developing lymphocyte lineages at the organ level.

## 12. Thymopoiesis in lampreys

The microenvironment of the thymoid of lampreys also expresses a *Foxn1*-like gene, which shares the conserved DNA binding domain and activation domain with the other members in the gene family, but has a longer N-terminal domain than Foxn1 of cartilaginous fishes; *Foxn1* gene expression co-localizes with the expression site of *DLL-B*, a putative Foxn1 target gene encoding a Delta-related Notch ligand [73]. The cells expressing *CDA1*, a potential marker of developing T cells and presumed to be required for *VLRA/VLRC* gene assembly, are located in close proximity of the *Foxn1* positive cells in the thymoids, strongly suggesting the presence of cellular interactions between epithelial cells and lymphoid precursors [73]. Non-functional *VLRA* and *VLRC* assemblies are detected in the thymoid region with higher frequency than in the periphery [24,73,88], compatible with the view that the thymoid is the site of VLRA/VLRC repertoire selection. However, no information is available about the magnitude and the underlying mechanism(s) of quality control in the thymoid. Interestingly, the *VLRB*-expressing B-like cells are absent from the thymoid region and their development appears to take place in the kidney and/or typhlosole [22,73]. This observation suggests that the anatomical segregation of the developmental sites for the two principal lymphoid lineages has a deep origin in the evolutionary history of the two adaptive immune systems in vertebrates. In the context of the reconstruction strategy described above, one may predict that the expression of lamprey *Foxn1* in the mouse thymus would support a T cell-biased lymphopoietic environment, with little if any concomitant B cell development.

## 13. Outlook

The evolutionary dynamics of the thymic microenvironment provides an instructive example for the role of external cues in imposing lineage decisions on haematopoietic progenitor cells. The lessons learned from the *in vivo* reconstructions of ancient forms of microenvironments indicate the presence of previously unrecognized checkpoints in the differentiation of TECs, which complement current efforts in characterizing subsets of TECs [89–92]. Moreover, the various types of thymic microenvironments formed by expression of different members of the *Foxn1/4* transcription factor gene family suggest a plausible sequence of events characterizing the lymphopoietic capacities of the thymopoietic microenvironment during the early stages of vertebrate evolution. It is to be expected that similar analyses will be informative with respect to the function of the microenvironment of lampreys, which are not easily amenable to genetic interrogation. Moreover, it will be interesting to include *Foxn1/4* gene family members of hagfishes into such comparative studies, since very little is known about the immune system of this second clade of jawless vertebrates.

## Data accessibility

This article has no additional data.
